# Incidence of Acute Gastroenteritis and Role of Norovirus, Georgia, USA, 2004–2005

**DOI:** 10.3201/eid1708.101533

**Published:** 2011-08

**Authors:** Aron J. Hall, Mariana Rosenthal, Nicole Gregoricus, Sharon A. Greene, Jeana Ferguson, Olga L. Henao, Jan Vinjé, Ben A. Lopman, Umesh D. Parashar, Marc-Alain Widdowson

**Affiliations:** Author affiliations: Centers for Disease Control and Prevention, Atlanta, Georgia, USA (A.J. Hall, M. Rosenthal, N. Gregoricus, S.A. Greene, O.L. Henao, J. Vinjé, B.A. Lopman, U.D. Parashar, M.-A. Widdowson);; University of Michigan, Ann Arbor, Michigan, USA (M. Rosenthal);; Kaiser Permanente, Atlanta (J. Ferguson)

**Keywords:** norovirus, viruses, gastroenteritis, AGE, etiology, incidence, Georgia, United States, research

## Abstract

Approximately 179 million cases of acute gastroenteritis (AGE) occur annually in the United States. However, lack of routine clinical testing for viruses limits understanding of their role among persons seeking medical care. Fecal specimens submitted for routine bacterial culture through a health maintenance organization in Georgia, USA, were tested with molecular diagnostic assays for norovirus, rotavirus, astrovirus, sapovirus, and adenovirus. Incidence was estimated by using national health care utilization rates. Routine clinical diagnostics identified a pathogen in 42 (7.3%) of 572 specimens; inclusion of molecular viral testing increased pathogen detection to 15.7%. Community AGE incidence was 41,000 cases/100,000 person-years and outpatient incidence was 5,400/100,000 person-years. Norovirus was the most common pathogen, accounting for 6,500 (16%) and 640 (12%) per 100,000 person-years of community and outpatient AGE episodes, respectively. This study demonstrates that noroviruses are leading causes of AGE among persons seeking medical care.

## Abstract 2

TOC summary: Improved clinical assays will guide appropriate case management.

Acute gastroenteritis (AGE), defined as diarrheal disease of rapid onset potentially accompanied by nausea, vomiting, fever, or abdominal pain, is a major cause of illness in the United States. Approximately 179 million episodes of AGE occur each year and result in ≈600,000 hospitalizations and 5,000 deaths (*1**,**2*). A specific etiology is attributed to only ≈20% of AGE cases, although viruses are recognized as the most common of the known agents (*1**,**3*). Noroviruses, in particular, have been estimated to cause ≈21 million cases of AGE annually, including >56,000 hospitalizations and 570 deaths (*3*). However, these estimates are based on US estimates of AGE and extrapolation of etiologic fractions from studies in other industrialized countries because few laboratory-based data are available on the role of noroviruses in sporadic AGE in the United States.

Development of more precise disease incidence estimates for noroviruses and other viral causes of AGE have been hampered, in part, by the lack of diagnostic assays available in clinical settings. With the exception of an enzyme immunoassay (EIA) for rotavirus, diagnosis of viral AGE in the United States is made largely on the basis of clinical signs and symptoms. Molecular techniques used for definitive diagnosis, specifically PCR, are available mostly in public health laboratories and research settings. Commercial EIA kits for norovirus have been developed but are not widely available in the United States and are not cleared by the US Food and Drug Administration for diagnosis of sporadic AGE cases. Evaluation of viral AGE incidence is further limited by the fact that most AGE patients do not seek medical care, and, of those who do, <20% submit fecal specimens for diagnostics (*2*). Lastly, general perception is that norovirus gastroenteritis is a self-limiting mild illness that rarely requires medical attention, despite several reports of serious illness and death in various settings (*3**–**7*).

Better understanding of the relative role of specific viral causes of AGE among persons seeking medical care is needed to help guide clinical management and ultimately to develop more appropriate AGE prevention strategies. We sought to determine the prevalence of viral pathogens among AGE patients who sought medical care and identify their trends in seasonality and molecular epidemiology. Using a similar strategy as that used for common bacterial and parasitic causes of AGE (*3*), we further sought to estimate incidence of viral agents of AGE in community and outpatient settings.

## Methods

### Study Population and Sample Selection

Kaiser Foundation Health Plan of Georgia, Inc. (KP) is a health maintenance organization with ≈280,000 members in Georgia, USA, who almost exclusively seek care with KP. One microbiology laboratory serves the entire population of Georgia KP members and receives ≈140 fecal specimens each month from outpatients seeking medical care. Upon order of fecal diagnostics by an outpatient clinician, patients were provided fecal collection kits and instructions for their in-home collection. Patients were instructed to keep specimens refrigerated after collection and to return them as soon as possible to the clinic, typically within 48 hours. Specimens were then transported by same-day courier to the KP microbiology laboratory for processing.

Each week during March 15, 2004–March 13, 2005, a total of 11 specimens from different patients were randomly selected for inclusion in the study. A target sample size of 572 was selected to identify 10% norovirus prevalence with a 95% confidence interval of ± 5%, assuming patients with AGE of short duration were 4× less likely to submit samples than those with AGE of longer duration. Only patients for whom a specimen was submitted after a clinician order for routine culture were eligible for inclusion, although additional diagnostics (e.g., ova and parasites, *Clostridium difficile*, rotavirus EIA) may have also been ordered for some patients. The following data were obtained for each specimen: days from outpatient visit to receipt at the KP laboratory, week of receipt by the KP laboratory, patient age group in 5-year intervals, patient sex, fecal consistency, and results of any diagnostic tests performed by the KP laboratory. Data and specimens sent to the Centers for Disease Control and Prevention (CDC), Atlanta, GA, USA, were anonymous and had no identifiable information that could be linked to the patient. Thus, our study did not require review by an institutional review board.

### Laboratory Testing

All specimens submitted for routine culture were tested for *Campylobacter*, *Shigella*, and *Salmonella* spp. at the KP laboratory. When requested, specimens were also tested for *Giardia* and *Cryptosporidium* spp. by using ProSpec T Microplate Assays (Remel, Lenexa, KS, USA), for rotavirus by using the Immunocard STAT Rotavirus Test (Meridian Bioscience Inc., Cincinnati, OH, USA), and for *C*. *difficile* toxigenic strains by using the *C. difficile* Tox A/B 11 EIA (Wampole Laboratories LLC, Princeton, NJ, USA). Specimens submitted in Cary Blair medium (i.e., routine bacterial culture) then underwent molecular testing for norovirus, rotavirus, sapovirus, astrovirus and enteric adenovirus at the CDC laboratory.

Viral nucleic acid was extracted by using RNA spin columns (Omega Bio-Tek, Doraville, GA, USA). TaqMan real-time reverse transcription PCR and PCR were used for initial sample screening for genogroup I (GI) and GII noroviruses, sapoviruses, and adenoviruses, as described (*8**–**10*). Conventional PCR was conducted to screen samples for astrovirus and rotavirus group A and to determine the genotype of norovirus-, sapovirus-, and adenovirus-positive samples (*11**–**15*). PCR products were purified by using the QIAquick Gel Extraction Kit (QIAGEN, Valencia, CA, USA) and sequenced by using the BigDye Terminator Cycle Sequencing Ready Reaction Kit (Applied Biosystems, Foster City, CA, USA) according to the manufacturers’ instructions. For genotyping, detected virus sequences were compared with sequences in the CDC reference sequence databases.

### Disease Incidence Calculations

The prevalence of each pathogen in specimens was used to calculate pathogen-specific incidence rates of acute gastroenteritis among the study population. We used pooled data on self-reported health care utilization practices of persons with acute diarrheal disease obtained from Foodborne Diseases Active Surveillance Network (FoodNet) population surveys in 2000–2001, 2002–2003 (*2*), and 2006–2007 (CDC, unpub. data). These 3 population-based telephone surveys were conducted in selected sites located throughout the United States, including Georgia, by using a probability sample design.

The weighted proportion of survey respondents with AGE (defined as diarrhea [>3 loose stools in a 24-hour period] beginning within the past month) who sought medical care in person and the proportion of those respondents who submitted a specimen were calculated and stratified by age group (Table 1). Because viral diarrhea is generally of shorter duration than diarrhea of other etiologies (*4*), data from respondents with diarrhea lasting <3 days at the time of interview were used for estimates of viral etiologies, and data from respondents with diarrhea of any duration were used for estimates of bacterial, parasitic, and other etiologies. These data were used to generate age-group–specific rates of fecal specimen submission among all those in the community with diarrhea and among only those seeking medical care. Respondents with a chronic disease for which diarrhea is a major sign (e.g., celiac disease, Crohn disease, diverticulitis, irritable bowel syndrome, ulcerative colitis) were excluded from analysis.

**Table 1:** Health care utilization practices among persons with acute gastroenteritis, by age group, FoodNet Population Surveys, USA, 2000–2007*

Age group, y	% Persons with acute gastroenteritis who sought medical care			% Persons who submitted a fecal specimen of those with acute gastroenteritis who sought medical care	
	<3 d illness duration	Any illness duration		<3 d illness duration	Any illness duration
<5	16.8	28.5		5.8	15.8
5–15	16.8	20.1		6.9	10.9
16–25	6.2	12.4		1.6	0.6
26–45	6.4	10.7		1.5	13.7
46–65	5.4	9.5		7.5	21.5
>65	10.3	15.7		17.4	13.0
Total	9.3	14.6		6.1	13.3

*Acute gastroenteritis is defined as diarrhea (>3 loose stools in a 24-h period) beginning within the past month and in the absence of a chronic disease for which diarrhea is a major sign. Data were obtained from the Foodborne Diseases Active Surveillance Network (FoodNet) Population Survey, cycles 3–5 (*2*; Centers for Disease Control and Prevention, unpub. data).

**Figure 1:**
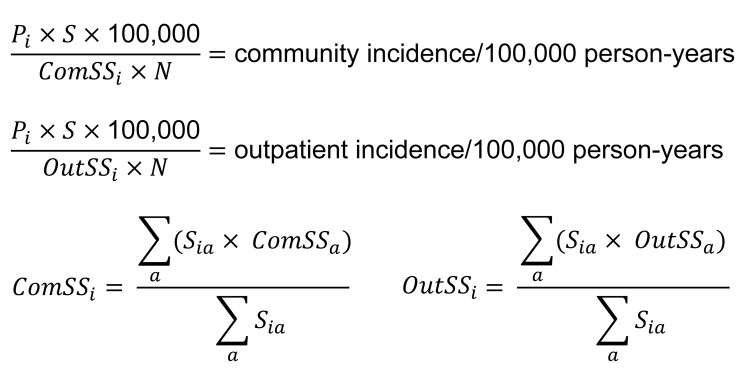
Equations used for calculation of community and outpatient incidence of each pathogen in patients with acute gastroenteritis (AGE), Kaiser Foundation Health Plan of Georgia, Inc., USA, March 15, 2004–March 13, 2005. *P_i_*, prevalence of pathogen *i* in stool samples; *S*, total no. stool samples submitted; *ComSS_i_*, pathogen-specific fecal specimen submission rates among all respondents with AGE; *N*, total Kaiser membership; *OutSS_i_*, pathogen-specific fecal specimen submission rates among those seeking medical care; *S_ia_*, no. of stools positive for pathogen *i* in age group *a*; *ComSS_a_*, proportion of those in age group *a* with AGE who seek care and submit stool specimen; *OutSS_a_*, proportion of those in age group *a* seeking care for AGE who submit stool specimen.

To adjust for variations in health care utilization practices by age, we weighted age-group–specific FoodNet Population Survey data proportional to the age distribution of persons with specimens positive for each pathogen by using the following age groups: <5, 5–15, 16–25, 26–45, 46–65, and >65 years. These age groups were broadly selected for clinical relevance and consistency in health care utilization rates. Community and outpatient incidence of each pathogen was calculated on the basis of prevalence of that pathogen in sampled specimens (*P_i_*), pathogen-specific fecal specimen submission rates among all respondents with AGE (*ComSS_i_*) and among those seeking medical care (*OutSS_i_*), the total number of specimens submitted to the KP laboratory during the study period (*S* = 1,825), and the total Kaiser membership in Georgia (i.e., study catchment population, N = 280,000) (Figure 1).

Thus, incidence was calculated on the basis of extrapolation of pathogen prevalence by using fecal specimen submission and medical care–seeking rates for community estimates and only fecal specimen submission rates for outpatient estimates. A simulation approach was used to generate 90% credible intervals (CIs). For each pathogen or group of pathogens, *P_i_*, *ComSS_i_*, and *OutSS_i_* were randomly drawn assuming a β distribution for each, and the 2 incidence equations were recalculated. We report 5th and 95th centiles of 100,000 simulations. Incidence estimates weighted for monthly fluctuations in the number of specimens received by the KP laboratory were also evaluated, but because they did not differ appreciably with unadjusted estimates, the simpler unadjusted approach was used. Chi-square tests were used to evaluate trends among categorical variables. Analyses were performed by using SAS version 9.2 (SAS Institute, Cary, NC, USA), Stata version 11.0 (StataCorp LP, College Station, TX, USA), and Epi Info version 3.4.3 (CDC).

## Results

A total of 572 specimens were included in the study. Routine bacterial culture and viral PCR were performed on all specimens, and 375 (65.6%) were also tested for ova and parasites, 161 (28.1%) for *C. difficile*, and 28 (4.9%) for rotavirus by using EIA. Specimens not tested for ova and parasites or *C. difficile* were considered not positive for those pathogens. Adult patients 26–65 years of age provided 325 (56.8%) of the specimens tested and 316 (55.2%) were from female patients. Clinical diagnostic testing at the KP laboratory identified a pathogen in 42 (7.3%) specimens. Subsequent PCR testing at CDC identified >1 virus in 53 (9.3%) specimens, for a total of 88 (15.4%) specimens with a detected pathogen (includes 5 specimens previously positive for rotavirus by EIA and 2 specimens positive for bacteria and virus). Among these 88 specimens, viruses were detected in 53 (60.2%), bacteria in 30 (34.1%), and parasites in 7 (8.0%). Multiple pathogens were identified in only 4 specimens (Table 2). Norovirus was the most frequently identified pathogen overall, detected in 25 (4.4%) of all specimens and 28.4% of the 88 specimens with any pathogen identified. Detection of any pathogen in feces was most likely in children <5 years of age (32.1%) and decreased significantly with age to 3.6% for persons >65 years of age (p<0.001).

**Table 2:** Pathogens detected among fecal specimens submitted by outpatients, by age group, to Kaiser Foundation Health Plan of Georgia, Inc., USA, March 15, 2004–March 13, 2005

Pathogen	Age group, y, no. (%) positive						Total, n = 572
	<5, n = 81	5–15, n = 63	16–25, n = 47	26–45, n = 190	46–65, n = 135	>65, n = 56	
Virus	19 (23.5)	5 (7.9)	6 (12.8)	14 (7.4)	7 (5.2)	2 (3.6)	53 (9.3)
Norovirus	6 (7.4)	1 (1.6)	3 (6.4)	8 (4.2)	7 (5.2)	0	25 (4.4)
Astrovirus	5 (6.2)	1 (1.6)	1 (2.1)	3 (1.6)	0	0	10 (1.7)
Rotavirus	4 (4.9)	2 (3.2)	1 (2.1)	0	0	0	7 (1.2)
Sapovirus	4 (4.9)	0	1 (2.1)	0	1 (0.7)	1 (1.8)	7 (1.2)
Adenovirus	1 (1.2)	1 (1.6)	0	3 (1.6)	0	1 (1.8)	6 (1.0)
Bacteria	8 (9.9)	3 (4.8)	3 (6.4)	8 (4.2)	8 (5.9)	0	30 (5.2)
Clostridium difficile*	0	0	1 (2.1)	6 (3.2)	7 (5.2)	0	14 (2.4)
*Salmonella* spp.	6 (7.4)	1 (1.6)	1 (2.1)	0	0	0	8 (1.4)
*Shigella* spp.	2 (2.5)	1 (1.6)	0	2 (1.1)	0	0	5 (0.9)
*Campylobacter* spp.	0	1 (1.6)	1 (2.1)	0	1 (0.7)	0	3 (0.5)
Parasite†	1 (1.2)	1 (1.6)	1 (2.1)	4 (2.1)	0	0	7 (1.2)
*Giardia* spp.	0	1 (1.6)	0	4 (2.1)	0	0	5 (0.9)
*Cryptosporidium* spp.	1 (1.2)	1 (1.6)	0	0	0	0	2 (0.3)
Multiple‡	3 (3.7)	0	0	0	1 (0.7)	0	4 (0.7)
Any pathogen	26 (32.1)	9 (14.3)	10 (21.3)	26 (13.7)	15 (11.1)	2 (3.6)	88 (15.4)
Unidentified	55 (67.9)	54 (85.7)	37 (78.7)	164 (86.3)	120 (88.9)	54 (96.4)	484 (84.6)

*C. difficile testing performed on 161 (28.1%) of 572 total specimens; those not tested were classified as not positive. †Parasite testing performed on 375 (65.6%) of 572 total specimens; those not tested were classified as not positive. ‡Combinations observed were *Salmonella* spp./norovirus, *Salmonella* spp./sapovirus, norovirus/sapovirus, and rotavirus/sapovirus.

Most (62.4%) specimens were received for laboratory testing within 3 days of the patient’s medical visit. However, 90 (15.7%) were received >1 week later. Time lag did not differ by age group. Pathogens were detected most frequently among specimens received by the laboratory within 3 days of outpatient visit (18.2%) and were significantly less likely to be detected with increasing delay, decreasing to 6.7% among specimens received after >1 week (p<0.001). This time lag effect was more pronounced for viruses than bacteria and was likely caused by the decrease in viral shedding and test sensitivity after the acute phase of illness. For example, norovirus was identified in 5.0% of specimens received <7 days of outpatient visit, but in only 1.1% of those received >1 week after outpatient visit; overall detection of bacteria decreased from 5.6% to 3.3% during the same time frame. Most (81.4%) specimens were unformed (i.e., took on the shape of the collection cup), indicating that the patients were still symptomatic at the time of specimen collection. No differences in rates of pathogen detection were found between formed and unformed specimens.

**Figure 2:**
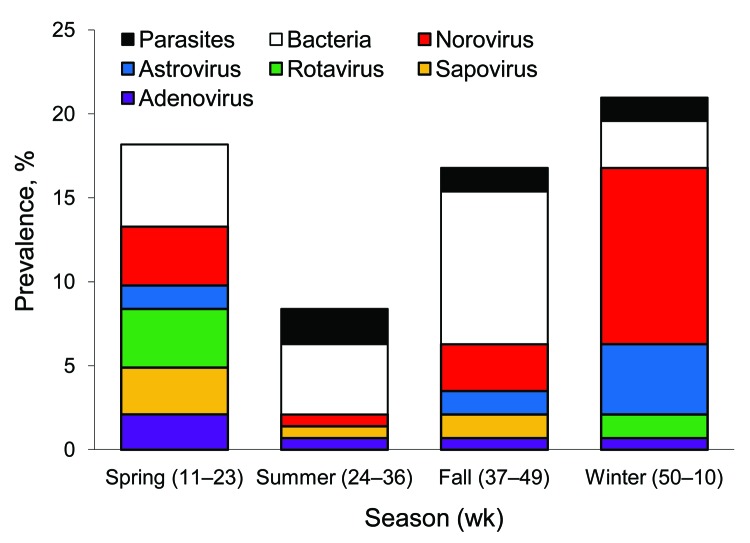
Pathogen prevalence in acute gastroenteritis outpatients by season, Kaiser Foundation Health Plan of Georgia, Inc., USA, March 15, 2004–March 13, 2005. Viral pathogens predominated during winter and spring, and bacteria predominated during summer and fall.

Overall rate of pathogen detection and the relative distribution of pathogens showed apparent seasonal variation, although insufficient sample size precluded identification of significant temporal trends. Overall, viruses predominated during winter and spring, and bacteria predominated during summer and fall (Figure 2). Relatively low prevalence of parasites was observed year round with no discernible seasonal pattern. Pronounced winter peaks in prevalence were observed for norovirus (10.5%) and astrovirus (4.2%). All rotavirus-positive specimens were received during winter and spring, during which prevalence was 1.4% and 3.5%, respectively. Similarly, peak prevalence of sapovirus (2.8%) and adenovirus (2.1%) was during spring.

Overall estimated community incidence of AGE of any etiology was 41,000 cases/100,000 person-years (90% CI 38,000–44,000 cases), of which 13,000 (32%, 90% CI 10,000–20,000 cases) were caused by identified agents (Table 3). Estimated incidence of AGE in outpatients was 5,400 cases/100,000 person-years (90% CI 4,400–6,700 cases), of which 1,600 (30%, 90% CI 1,300–2,400 cases) could be attributed to a specific pathogen. Norovirus was the leading identifiable cause of illness in community and outpatient settings and was associated with 16% of all community AGE (6,500 cases/100,000 person-years, 90% CI 3,700–12,000 cases) and 12% of all outpatient AGE (640/100,000 person-years, 90% CI 360–1,200 cases).

**Table 3:** Estimated incidence of pathogens causing acute gastroenteritis in community and outpatient settings, Kaiser Foundation Health Plan of Georgia, Inc., USA, March 15, 2004–March 13, 2005*

Pathogen	Outpatient†			Community‡	
	Incidence (90% CI)§	% Total		Incidence (90% CI)§	% Total
Virus	1,300 (750–2,200)	24.1		11,000 (6,800–19,000)	26.8
Norovirus	640 (360–1,200)	11.9		6,500 (3,700–12,000)	15.9
Astrovirus	270 (130–590)	5.0		1,800 (880–3,400)	4.4
Rotavirus	150 (65–330)	2.8		880 (400–1,700)	2.1
Sapovirus	110 (49–220)	2.0		900 (420–1,800)	2.2
Adenovirus	120 (49–260)	2.2		970 (410–2,100)	2.4
Bacteria	240 (160–320)	4.4		1,700 (1,100–2,300)	4.1
* Clostridium difficile*	96 (61–150)	1.8		960 (590–1,500)	2.3
*Salmonella* spp.	69 (35–120)	1.3		250 (130–440)	0.6
*Shigella* spp.	41 (17–85)	0.8		200 (86–410)	0.5
*Campylobacter* spp.	31 (9–76)	0.6		240 (71–590)	0.6
Parasite	60 (29–110)	1.1		420 (200–790)	1.0
*Giardia* spp.	43 (18–86)	0.8		350 (150–720)	0.9
*Cryptosporidium* spp.	17 (4–48)	0.3		68 (14–190)	0.2
Any pathogen	1,600 (1,300–2,400)	29.6		13,000 (10,000–20,000)	31.7
Unidentified	3,800 (3,200–4,600)	70.4		28,000 (23,000–34,000)	68.3
Total	5,400 (4,400–6,700)	100		41,000 (38,000–44,000)	100

*Incidence is per 100,000 person-years. Acute gastroenteritis is defined as diarrhea (>3 loose stools in a 24-h period) beginning within the past month and in the absence of a chronic disease for which diarrhea is a major sign. CI, credible interval. †Outpatient incidence calculated from prevalence in fecal specimens sampled, age-adjusted fecal specimen submission rates among health care seekers, number of fecal specimens submitted to the Kaiser laboratory annually (1,825), and number of Kaiser memberships in Georgia (280,000). ‡Community incidence calculated from prevalence in fecal specimens sampled, age-adjusted medical care seeking and fecal specimen submission rates, number of fecal specimens submitted to the Kaiser laboratory annually (1,825) and total Kaiser memberships in Georgia (280,000). §90% CI calculated from the 5th and 95th percentiles of 100,000 simulations, assuming a β distribution of variables.

In comparison with estimated community incidence of AGE caused by bacteria (1,700 cases/100,000 person-years, 90% CI 1,100–2,300) and parasites (420 cases/100,000 person-years, 90% CI 200–790), the community incidence of norovirus was ≈4-fold and >15-fold greater, respectively. Likewise, the incidence of norovirus infection prompting medical care was ≈3-fold greater than that of bacterial pathogens (240 cases/100,000 person-years, 90% CI 160–320 cases) and >10-fold greater than that of parasites (60 cases/100,000 person-years, 90% CI 29–110 cases). Astrovirus was the second leading identifiable pathogen associated with AGE in community and outpatient settings; estimated incidences were 1,800 cases (90% CI 880–3,400 cases) and 270 (90% CI 130–590 cases) per 100,000 person-years, respectively.

**Figure 3:**
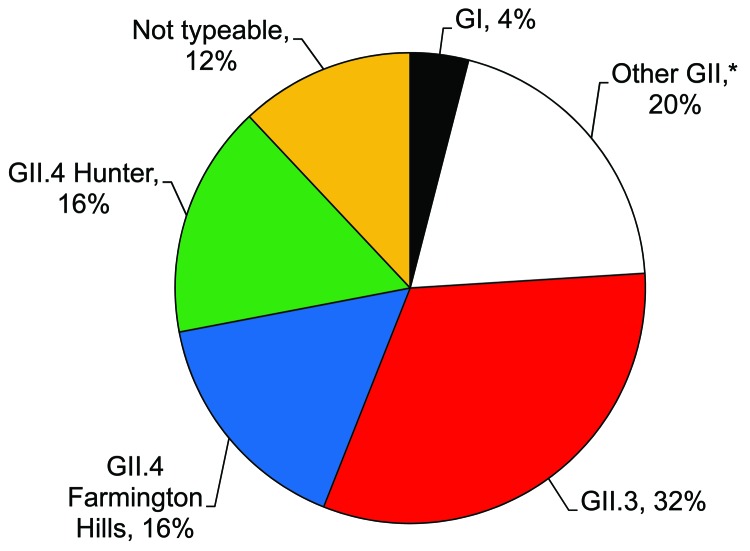
Distribution of norovirus genotypes among 25 outpatients with acute gastroenteritis, Kaiser Foundation Health Plan of Georgia, Inc., USA, March 15, 2004–March 13, 2005. Genogroup II (GII) was more prevalent than GI. *Includes GII.2 (2 specimens), GII.14 (2 specimens), and GII.17 (1 specimen).

Sequence analysis of the 25 norovirus-positive samples identified 24 (96%) as GII strains and 1 (4%) as a GI strain (Figure 3). Of all strains, approximately one third could be typed as GII.4, specifically the Farmington Hills and Hunter subclusters. GII.3 viruses were equally prevalent in this population. Other norovirus genotypes identified included GI3b, GII.2, GII.14, and GII.17.

## Discussion

This study provides US laboratory–based incidence rates for norovirus disease determined by using direct outpatient surveillance of routinely collected fecal specimens. Despite examination of only fecal specimens submitted for bacterial culture, our data demonstrate that viruses, and noroviruses specifically, were the leading cause of AGE among persons of all ages seeking medical care. We estimate that 24% of persons seeking medical care for AGE do so because of viral infection, including 12% because of norovirus infection. Overall, viruses accounted for 27% of AGE episodes in the community and noroviruses accounted for 16%.

In contrast to bacterial and parasitic pathogens, viral AGE pathogens were primarily detected during winter and spring months, which is consistent with previous descriptions of norovirus and rotavirus seasonality in temperate climates (*16*). Norovirus strains identified in this study were similar to those identified in studies of sporadic AGE (*17*) and those implicated in norovirus outbreaks confirmed by CDC during the same period (*18*). The predominance of GII.4 strains in epidemic and sporadic norovirus disease demonstrates the need for including this genotype in vaccine development efforts.

Our findings are consistent with those of studies conducted in other industrialized countries, which demonstrated the relative role of viral pathogens in causing AGE (*19**–**23*). A recent systematic literature review that included 13 etiologic studies of community and outpatient cases of sporadic diarrhea, most of which focused on children, determined that norovirus was responsible for 12% (range 5%–36%) of AGE cases (*17*). In a community-based study in Germany, the incidence of AGE requiring medical consultation attributable to norovirus was 626 cases/100,000 person-years (*19*). In England, the incidence of general practice consultations for norovirus-associated infectious intestinal disease was 540 cases/100,000 person-years (*24*). The estimated incidence of norovirus among outpatients in our study (640 cases/100,000 person-years) supports the findings of the studies in England and Germany. A similar study in the Netherlands reported a considerably lower incidence of gastroenteritis in general practices (797 cases/100,000 person-years), in which 5% (40 cases/100,000 person-years) were caused by norovirus (*20*). However, standardized gastroenteritis incidence reported in the Dutch community of 28,300 cases/100,000 person-years is more similar to that estimated in our study (41,000 cases/100,000 person-years) (*25*). Dutch guidelines for general practitioners, which recommend that cases of uncomplicated AGE be handled by telephone consultation (http://nhg.artsennet.nl), may explain the apparent difference in health care utilization practices.

Prior US estimates of the incidence of norovirus have been based on a bottom-up approach of using overall incidence of AGE from population surveys and estimating the proportion attributable to norovirus on the basis of data from other countries, given the lack of laboratory-based data available at the time (*3*). This approach yielded an estimate of ≈21 million cases of norovirus illness annually, corresponding to a community incidence similar to that estimated in our study (6,974 cases/100,000 person-years vs. 6,500 cases/100,000 person-years). Similarly generated bottom-up estimates for astrovirus, rotavirus, and sapovirus (each 1,024 cases/100,000 person-years) were likewise within 90% CIs of our community incidence estimates (*3*).

Although markedly different approaches were used, each subject to considerable uncertainty, concordance of the respective incidence estimates generated is reassuring. However, given the inclusion of only specimens submitted for routine bacterial diagnostics, our study likely underestimated the true incidence of norovirus disease in the community. Furthermore, use of health care utilization multipliers based primarily on diarrhea may underestimate the incidence of pathogens that can cause other AGE signs (e.g., vomiting) in the absence of diarrhea, as has been reported for norovirus (*26*). Previous studies have demonstrated that seeking medical care is influenced by disease severity and social factors (*27**,**28*), which leads to a smaller proportion of viral AGE patients in the community who seek medical care, compared with bacterial and parasitic AGE (*22*). Identifying laboratory-confirmed cases of norovirus infection and extrapolating community incidence by using pathogen-specific health care utilization multipliers in a top-down approach may be preferable when such data are available and would likely yield more reliable estimates.

The primary limitation of this study results from use of routinely collected specimens, as opposed to systematic, active patient recruitment. The study specimens may have therefore been obtained from patients with noninfectious or chronic diseases, which may have contributed to the low detection rate for pathogens in this study. Delays in collection of specimens may have also contributed to the low rates of pathogen detection. Routinely collected samples also overrepresent some age groups, as demonstrated by differential fecal submission rates (Table 1), which may not reflect age groups at greatest risk for infection by specific pathogens or risk for the overall population.

We have adjusted for this sampling artifact by development of pathogen-specific, age-adjusted health care utilization multipliers. These multipliers resulted in the observed increase from pathogen prevalence in specimens (e.g., 4.4% for norovirus) to pathogen prevalence among estimated community AGE cases (e.g., 16% for norovirus). In addition to age, pathogen-specific health care utilization multipliers also accounted for duration of illness and yielded different pathogen distributions in community and outpatient settings. These results are consistent with those of previous studies (*20**,**22**,**25*). In contrast to a previous study that estimated US incidence of AGE (*3*), our study did not adjust for differential health care utilization rates based on illness severity (i.e., bloody vs. nonbloody diarrhea) to maintain adequate power to enable the adjustment for age group. Nonetheless, there was insufficient sample size within each age group to generate age group–specific disease incidence estimates.

Attributing causality of AGE to norovirus infection is complicated by the fact that asymptomatic infections may occur; healthy persons used in previous studies as controls have shown background rates of infection of 1%–16% (*17*). However, low prevalence (1.1%) of norovirus in specimens received >1 week after outpatient visit and low number of mixed infections identified in our study suggest that norovirus was likely the etiologic agent when detected. Incidence estimates were not adjusted for test sensitivity because the molecular methods used for viral diagnostics generally show extremely high sensitivities if appropriate and timely specimens are tested. Prevalence and incidence of parasites and *C. difficile* may be underestimated given that not all specimens were tested for these pathogens and indications for clinicians requesting specific tests are unknown and likely varied. Finally, given that the study population included only Georgia residents and health maintenance organization members, which tends toward younger age and higher socioeconomic status, the results may not be generalizable to the overall US population. Restriction of FoodNet data to only respondents in Georgia who had health insurance was evaluated for comparison but did not differ. Therefore, all respondents were ultimately included to enable adequate power for age stratification.

Development of sensitive clinical assays for identification of viral agents of AGE, such as norovirus, and more widespread use of such assays may help close the diagnostic gap on sporadic AGE cases and guide more appropriate case management. The demonstrated predominance of viruses among medically attended AGE cases should help prevent the unnecessary use of antimicrobial drugs and spur development of novel interventions specific for the unique transmission pathways of viruses. Compared with bacterial AGE etiologies, many of which result from foodborne transmission from infected animal sources, viral AGE pathogens originate in human reservoirs and usually involve direct or indirect person-to-person spread. Although occasionally implicated in outbreaks (*29**,**30*), the role of foods contaminated during processing in the overall norovirus disease incidence remains largely unknown. Further assessment of the incidence of enteric viruses, including hospital-based studies of risk factors for severe disease and attribution to specific transmission pathways, are needed to improve control measures and assess future potential of vaccines.
